# West Nile Virus and Other Nationally Notifiable Arboviral Diseases — United States, 2017

**DOI:** 10.15585/mmwr.mm6741a1

**Published:** 2018-10-19

**Authors:** Emily J. Curren, Jennifer Lehman, Jonathan Kolsin, William L. Walker, Stacey W. Martin, J. Erin Staples, Susan L. Hills, Carolyn V. Gould, Ingrid B. Rabe, Marc Fischer, Nicole P. Lindsey

**Affiliations:** ^1^Epidemic Intelligence Service, CDC; ^2^Arboviral Diseases Branch, Division of Vector-Borne Diseases, National Center for Emerging and Zoonotic Infectious Diseases, CDC.

Arthropodborne viruses (arboviruses) are transmitted to humans primarily through the bites of infected mosquitoes or ticks. West Nile virus (WNV) is the leading cause of domestically acquired arboviral disease in the continental United States (*1*). Other arboviruses, including Jamestown Canyon, La Crosse, Powassan, St. Louis encephalitis, and eastern equine encephalitis viruses, cause sporadic cases of disease and occasional outbreaks. This report summarizes surveillance data reported to CDC from U.S. states in 2017 for nationally notifiable arboviruses. It excludes dengue, chikungunya, and Zika viruses because, in the continental United States, these viruses are acquired primarily through travel. In 2017, 48 states and the District of Columbia (DC) reported 2,291 cases of domestic arboviral disease, including 2,097 (92%) WNV disease cases. Among the WNV disease cases, 1,425 (68%) were classified as neuroinvasive disease (e.g., meningitis, encephalitis, or acute flaccid paralysis), for a national rate of 0.44 cases per 100,000 population. More Jamestown Canyon and Powassan virus disease cases were reported in 2017 than in any previous year. Because arboviral diseases continue to cause serious illness, maintaining surveillance is important to direct and promote prevention activities.

Arboviruses are maintained in transmission cycles between arthropods and vertebrate hosts, including humans and other animals (*2*). Humans primarily become infected when bitten by an infected tick or mosquito. Most human infections are asymptomatic; symptomatic infections commonly manifest as a systemic febrile illness and less commonly as neuroinvasive disease.

Most endemic arboviral diseases are nationally notifiable and are reported by state health departments to CDC through ArboNET using standard surveillance case definitions that include clinical and laboratory criteria (*3*). Confirmed and probable cases are included in this analysis. Cases reported as meningitis, encephalitis, acute flaccid paralysis, or other neurologic illnesses are classified as neuroinvasive disease; the remainder are considered non-neuroinvasive disease. Incidence was calculated using neuroinvasive disease cases and the 2017 U.S. Census mid-year population estimates.

In 2017, 2,291 cases of domestic arboviral disease were reported to CDC; 1,596 (70%) were neuroinvasive. Cases were caused by the following viruses: WNV (2,097 cases; 92%), Jamestown Canyon (75), La Crosse (63), Powassan (34), St. Louis encephalitis (11), unspecified California serogroup (six), and eastern equine encephalitis (five). No cases were reported from Alaska or Hawaii. Among the 3,007 counties in the contiguous United States, 722 (24%) reported one or more arboviral disease cases.

Overall, 2,097 WNV disease cases were reported from 641 counties in 47 states and DC. Among these cases, 1,425 (68%) were neuroinvasive, and 1,814 (87%) patients had illness onset during July–September (Table 1). The median patient age was 59 years (interquartile range [IQR] = 46–69); 1,301 (62%) were male. A total of 1,545 (74%) patients were hospitalized, and 146 (7%) died. The median age of patients who died was 77 years (IQR = 68–84).

**TABLE 1:** Number and percentage of reported cases of West Nile virus and other arboviral diseases, by virus type and selected patient characteristics — United States, 2017

Characteristic	Virus no. (%)					
	West Nile* (N = 2,097)	Jamestown Canyon (N = 75)	La Crosse (N = 63)	Powassan (N = 34)	St. Louis encephalitis* (N = 11)	Eastern equine encephalitis (N = 5)
**Age group (yrs)**						
<18	50 (2)	4 (5)	54 (86)	5 (15)	0 (0)	0 (0)
18–59	1,020 (49)	36 (48)	6 (10)	10 (29)	5 (45)	5 (100)
≥60	1,027 (49)	35 (47)	3 (5)	19 (56)	6 (55)	0 (0)
**Sex**						
Male	1,301 (62)	46 (61)	34 (54)	28 (82)	8 (73)	2 (40)
Female	796 (38)	29 (39)	29 (46)	6 (18)	3 (27)	3 (60)
**Period of illness onset**						
January–March	7 (<1)	1 (1)	1 (2)	0 (0)	1 (9)	0 (0)
April–June	87 (4)	25 (33)	4 (6)	21 (62)	0 (0)	0 (0)
July–September	1,814 (87)	45 (60)	53 (84)	7 (21)	8 (73)	1 (20)
October–December	185 (9)	4 (5)	5 (8)	6 (18)	1 (9)	4 (80)
**Clinical syndrome**						
Nonneuroinvasive	672 (32)	17 (23)	0 (0)	1 (3)	5 (45)	0 (0)
Neuroinvasive						
Encephalitis	714 (34)	29 (39)	53 (84)	22 (65)	2 (18)	3 (60)
Meningitis	530 (25)	5 (7)	10 (16)	7 (21)	3 (27)	0 (0)
AFP	89 (4)	4 (5)	0 (0)	2 (6)	1 (9)	0 (0)
Other	92 (4)	20 (27)	0 (0)	2 (6)	0 (0)	2 (40)
**Outcome**						
Hospitalization	1,545 (74)	46 (61)	63 (100)	33 (97)	6 (55)	5 (100)
Death	146 (7)	2 (3)	0 (0)	2 (6)	0 (0)	2 (40)

**Abbreviation:** AFP = acute flaccid paralysis.

*Date of illness onset missing for four cases of West Nile virus and one case of St. Louis encephalitis virus.

Among the 1,425 WNV neuroinvasive disease cases, 714 (50%) were reported as encephalitis, 530 (37%) as meningitis, 89 (6%) as acute flaccid paralysis, and 92 (6%) as other neurologic illness. Among the 89 patients with acute flaccid paralysis, 34 (38%) also had encephalitis or meningitis. Among patients with neuroinvasive disease, 1,346 (94%) were hospitalized, and 146 (10%) died. The rate of neuroinvasive disease in the United States was 0.44 per 100,000 population (Table 2). The highest rates were in South Dakota (3.10 per 100,000), North Dakota (2.65), Mississippi (1.54), Arizona (1.40), and Utah (1.26) (Figure). The largest numbers of neuroinvasive disease cases were reported from California (401), Arizona (98), Texas (87), and Illinois (72), and together accounted for 46% of neuroinvasive disease cases. The rate of WNV neuroinvasive disease increased with patient age, from 0.02 per 100,000 in children aged <10 years to 1.28 in adults aged ≥70 years. The rate was higher among males (0.57 per 100,000) than among females (0.31).

**TABLE 2:** Number and rate* of reported cases of arboviral neuroinvasive disease, by virus type, U.S. Census division, and state — United States, 2017

U.S. Census Division/State	Virus											
	West Nile		Jamestown Canyon		La Crosse		Powassan		St. Louis encephalitis		Eastern equine encephalitis	
	No.	Rate	No.	Rate	No.	Rate	No.	Rate	No.	Rate	No.	Rate
United States	1,425	0.44	58	0.02	63	0.02	33	0.01	6	<0.01	5	<0.01
**New England**	**10**	**0.07**	**5**	**0.03**	**—^†^**	**—**	**9**	**0.06**	**—**	**—**	**—**	**—**
Connecticut	2	0.06	—	—	—	—	—	—	—	—	—	—
Maine	—	—	1	0.07	—	—	3	0.22	—	—	—	—
Massachusetts	5	0.07	2	0.03	—	—	3	0.04	—	—	—	—
New Hampshire	—	—	2	0.15	—	—	1	0.07	—	—	—	—
Rhode Island	1	0.09	—	—	—	—	2	0.19	—	—	—	—
Vermont	2	0.32	—	—	—	—	—	—	—	—	—	—
**Middle Atlantic**	**66**	**0.16**	**—**	**—**	**—**	**—**	**13**	**0.03**	**—**	**—**	**—**	**—**
New Jersey	6	0.07	—	—	—	—	4	0.04	—	—	—	—
New York	45	0.23	—	—	—	—	5	0.03	—	—	—	—
Pennsylvania	15	0.12	—	—	—	—	4	0.03	—	—	—	—
**East North Central**	**192**	**0.41**	**37**	**0.08**	**16**	**0.03**	**3**	**0.01**	**—**	**—**	**1**	**<0.01**
Illinois	72	0.56	—	—	1	0.01	—	—	—	—	—	—
Indiana	18	0.27	—	—	—	—	—	—	—	—	—	—
Michigan	32	0.32	—	—	—	—	—	—	—	—	—	—
Ohio	23	0.20	1	0.01	13	0.11	—	—	—	—	—	—
Wisconsin	47	0.81	36	0.62	2	0.03	3	0.05	—	—	1	0.02
**West North Central**	**118**	**0.55**	**14**	**0.07**	**2**	**0.01**	**8**	**0.04**	**—**	**—**	**—**	**—**
Iowa	10	0.32	—	—	1	0.03	—	—	—	—	—	—
Kansas	12	0.41	—	—	—	—	—	—	—	—	—	—
Minnesota	13	0.23	14	0.25	1	0.02	7	0.13	—	—	—	—
Missouri	17	0.28	—	—	—	—	—	—	—	—	—	—
Nebraska	19	0.99	—	—	—	—	—	—	—	—	—	—
North Dakota	20	2.65	—	—	—	—	1^§^	0.13	—	—	—	—
South Dakota	27	3.10	—	—	—	—	—	—	—	—	—	—
**South Atlantic**	**91**	**0.14**	**1**	**<0.01**	**28**	**0.04**	**—**	**—**	**—**	**—**	**4**	**0.01**
Delaware	—	—	—	—	—	—	—	—	—	—	—	—
District of Columbia	1	0.14	—	—	—	—	—	—	—	—	—	—
Florida	4	0.02	—	—	—	—	—	—	—	—	1	<0.01
Georgia	44	0.42	—	—	2	0.02	—	—	—	—	2	0.02
Maryland	5	0.08	—	—	1	0.02	—	—	—	—	1	0.02
North Carolina	8	0.08	1	0.01	21	0.20	—	—	—	—	—	—
South Carolina	16	0.32	—	—	—	—	—	—	—	—	—	—
Virginia	12	0.14	—	—	—	—	—	—	—	—	—	—
West Virginia	1	0.06	—	—	4	0.22	—	—	—	—	—	—
**East South Central**	**117**	**0.61**	**—**	**—**	**17**	**0.09**	**—**	**—**	**1**	**0.01**	**—**	**—**
Alabama	40	0.82	—	—	—	—	—	—	1	0.02	—	—
Kentucky	9	0.20	—	—	—	—	—	—	—	—	—	—
Mississippi	46	1.54	—	—	—	—	—	—	—	—	—	—
Tennessee	22	0.33	—	—	17	0.25	—	—	—	—	—	—
**West South Central**	**174**	**0.44**	**1**	**<0.01**	**—**	**—**	**—**	**—**	**—**	**—**	**—**	**—**
Arkansas	15	0.50	—	—	—	—	—	—	—	—	—	—
Louisiana	38	0.81	1	0.02	—	—	—	—	—	—	—	—
Oklahoma	34	0.86	—	—	—	—	—	—	—	—	—	—
Texas	87	0.31	—	—	—	—	—	—	—	—	—	—
**Mountain**	**243**	**1.01**	**—**	**—**	**—**	**—**	**—**	**—**	**3**	**0.01**	**—**	**—**
Arizona	98	1.40	—	—	—	—	—	—	3	0.04	—	—
Colorado	29	0.52	—	—	—	—	—	—	—	—	—	—
Idaho	16	0.93	—	—	—	—	—	—	—	—	—	—
Montana	3	0.29	—	—	—	—	—	—	—	—	—	—
Nevada	31	1.03	—	—	—	—	—	—	—	—	—	—
New Mexico	23	1.10	—	—	—	—	—	—	—	—	—	—
Utah	39	1.26	—	—	—	—	—	—	—	—	—	—
Wyoming	4	0.69	—	—	—	—	—	—	—	—	—	—
**Pacific**	**414**	**0.78**	**—**	**—**	**—**	**—**	**—**	**—**	**2**	**<0.01**	**—**	**—**
Alaska	—	—	—	—	—	—	—	—	—	—	—	—
California	401	1.01	—	—	—	—	—	—	2	0.01	—	—
Hawaii	—	—	—	—	—	—	—	—	—	—	—	—
Oregon	3	0.07	—	—	—	—	—	—	—	—	—	—
Washington	10	0.14	—	—	—	—	—	—	—	—	—	—

* Per 100,000 population, based on July 1, 2017, U.S. Census population estimates.

^†^ Dashes indicate none reported.

^§^ Patient reported travel to a state with a history of the virus.

**FIGURE:**
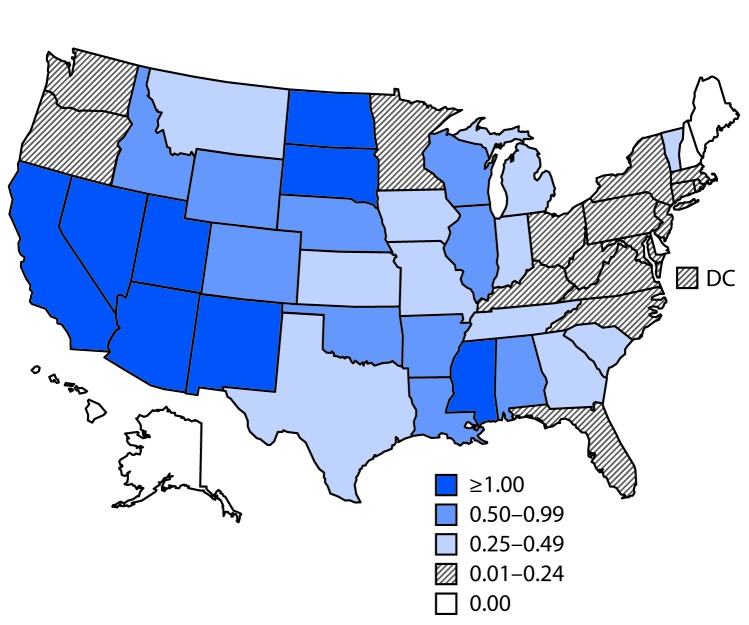
Rate* of reported cases of West Nile virus neuroinvasive disease — United States, 2017 * Per 100,000 population.

Seventy-five Jamestown Canyon virus disease cases were reported from eight states, primarily in the Northeast and upper Midwest (Table 2). In 2017, Jamestown Canyon virus disease was reported for the first time from Louisiana, Maine, and North Carolina. The median patient age was 58 years (IQR = 41–68), and 46 (61%) were male (Table 1). Illness onset ranged from January to November, with 45 (60%) patients reporting onset during July–September. Fifty-eight (77%) cases were neuroinvasive, 46 (61%) patients were hospitalized, and two (3%) died; both deaths were among patients aged ≥58 years with neuroinvasive disease. The rate of Jamestown Canyon virus neuroinvasive disease was highest in Wisconsin (0.62 per 100,000).

Sixty-three La Crosse virus disease cases were reported from 10 states, all in the Southeast and Midwest (Table 2). The median age of patients was 8 years (IQR = 5–12), and 54 (86%) were aged <18 years (Table 1). Illness onset dates ranged from March to October, with 53 (84%) reporting onset during July–September. All 63 cases were neuroinvasive and the patients were hospitalized; none died.

Thirty-four Powassan virus disease cases were reported from 10 states, primarily in the Northeast and Midwest (Table 2). The median patient age was 63 years (IQR = 48–74) and 28 (82%) were male (Table 1). Illness onset dates ranged from April to December, with 21 (62%) reporting onset during April–June. Powassan virus disease was reported for the first time from North Dakota in 2017, but the patient had history of travel to a state with previously documented transmission. Thirty-three (97%) cases were neuroinvasive, 33 (97%) patients were hospitalized (32 with neuroinvasive disease and one with non-neuroinvasive disease), and two (6%) patients died.

Eleven cases of St. Louis encephalitis virus disease were reported from three states (Alabama, Arizona, and California) (Table 2). The median patient age was 60 years (IQR = 48–63), and eight were male (Table 1). Illness onset dates ranged from January to October, with eight patients reporting onset during July–September. Six cases were neuroinvasive; all patients with neuroinvasive disease were hospitalized. No patients died.

Five cases of eastern equine encephalitis virus disease were reported from four states (Florida, Georgia, Maryland, and Wisconsin) (Table 2); however, infection occurred in three of the cases through organ transplantation. The median patient age was 42 years (IQR = 27–42), and two were male. All cases occurred in September and October. All cases were neuroinvasive, and the patients were hospitalized; two (40%) patients died.

## Discussion

As in previous years, WNV was the most common cause of neuroinvasive arboviral disease in the United States, accounting for 89% of reported neuroinvasive disease cases. The rate of WNV neuroinvasive disease in 2017 (0.44 per 100,000) was comparable to the median rate during 2007–2016 (0.41) (*4*). La Crosse virus continued to be the most common cause of neuroinvasive arboviral disease in children (*5*).

In 2017, eastern equine encephalitis virus transmission via organ transplantation was reported for the first time (*6*). More cases of Jamestown Canyon and Powassan virus disease were reported in 2017 than in any previous year: 75 Jamestown Canyon virus disease cases were reported in 2017 compared with a previous high of 16 cases in 2013, and 34 Powassan virus disease cases were reported in 2017 compared with a previous high of 22 cases in 2016 (*7*,*8*). These recent increases are likely caused by an increase in awareness and testing, but increased activity of these viruses cannot be ruled out. Deaths possibly associated with Jamestown Canyon virus infection are rare; however, two deaths were reported in 2017.

Arboviruses continue to cause substantial morbidity in the United States although reported numbers of cases vary annually. Cases occur sporadically, and the epidemiology varies by virus and geographic area. Consistent with previous years, in 2017, approximately 90% of arboviral disease cases occurred during April–September. Weather, zoonotic host and vector abundance, and human behavior are all factors that can influence when and where outbreaks occur. These factors make it difficult to predict future locations and timing of cases and emphasize the importance of surveillance to identify outbreaks and inform public health prevention efforts.

The findings in this report are subject to at least two limitations. First, because ArboNET does not require information about clinical signs and symptoms or laboratory findings, cases might be misclassified. Second, ArboNET is a passive surveillance system that only collects cases that are diagnosed and reported, resulting in underestimation of the actual incidence of disease. Detection and reporting of neuroinvasive disease are thought to be more consistent and more complete than they are for non-neuroinvasive disease. Previous studies have estimated that between 30 and 70 non-neuroinvasive disease cases occur for every reported case of WNV neuroinvasive disease (*9*). Based on the number of neuroinvasive disease cases reported in 2017, between 42,750 and 99,750 non-neuroinvasive disease cases of WNV would have been expected to occur; however, only 672 (1%–2%) were reported.

Health care providers need to consider arboviral infections in the differential diagnosis of aseptic meningitis and encephalitis, obtain appropriate specimens for laboratory testing, and promptly report cases to public health authorities (*2*,*3*). Understanding the epidemiology, seasonality, and geographic distribution of these viruses will assist with clinical recognition and differentiation from other neurologic infections. Because human vaccines against domestic arboviruses are not available, prevention depends on community and household efforts to reduce vector populations (e.g., applying insecticides and reducing breeding sites), personal protective measures to decrease exposure to mosquitoes and ticks (e.g., use of repellents and wearing protective clothing), and blood donor screening.

**Summary**

**What is already known about this topic?**

West Nile virus (WNV) is the leading cause of arboviral disease in the continental United States, but several other arboviruses cause sporadic cases and outbreaks of neuroinvasive disease.

**What is added by this report?**

In 2017, eastern equine encephalitis virus transmission via organ transplantation was reported for the first time. More cases of Jamestown Canyon and Powassan virus neuroinvasive disease were reported in 2017 than in any previous year.

**What are the implications for public health practice?**

Health care providers need to consider arboviral infections in the differential diagnosis of aseptic meningitis and encephalitis, obtain appropriate specimens for laboratory testing, and promptly report cases to public health authorities.
